# The role of post-translational modifications in driving abnormal cardiovascular complications at high altitude

**DOI:** 10.3389/fcvm.2022.886300

**Published:** 2022-09-14

**Authors:** Jun Hou, Xudong Wen, Pan Long, Shiqiang Xiong, Hanxiong Liu, Lin Cai, Haoyu Deng, Zhen Zhang

**Affiliations:** ^1^Department of Cardiology, Chengdu Third People’s Hospital, Cardiovascular Disease Research Institute of Chengdu, Affiliated Hospital of Southwest Jiaotong University, Chengdu, China; ^2^School of Material Science and Engineering, Southwest Jiaotong University, Chengdu, China; ^3^Department of Gastroenterology and Hepatology, Chengdu First People’s Hospital, Chengdu, China; ^4^Department of Medicine, Faculty of Medicine, University of British Columbia, Vancouver, BC, Canada; ^5^Center for Heart and Lung Innovation, St. Paul’s Hospital, University of British Columbia, Vancouver, BC, Canada; ^6^Department of Vascular Surgery, Renji Hospital, School of Medicine, Shanghai Jiao Tong University, Shanghai, China

**Keywords:** protein post-translational modifications (PTMs), hypobaric hypoxia, cardiovascular complications adverse events, high-altitude pulmonary edema (HAPE), lactylation modification

## Abstract

The high-altitude environment is characterized by hypobaric hypoxia, low temperatures, low humidity, and high radiation, which is a natural challenge for lowland residents entering. Previous studies have confirmed the acute and chronic effects of high altitude on the cardiovascular systems of lowlanders. Abnormal cardiovascular complications, including pulmonary edema, cardiac hypertrophy and pulmonary arterial hypertension were commonly explored. Effective evaluation of cardiovascular adaptive response in high altitude can provide a basis for early warning, prevention, diagnosis, and treatment of altitude diseases. At present, post-translational modifications (PTMs) of proteins are a key step to regulate their biological functions and dynamic interactions with other molecules. This process is regulated by countless enzymes called “writer, reader, and eraser,” and the performance is precisely controlled. Mutations and abnormal expression of these enzymes or their substrates have been implicated in the pathogenesis of cardiovascular diseases associated with high altitude. Although PTMs play an important regulatory role in key processes such as oxidative stress, apoptosis, proliferation, and hypoxia response, little attention has been paid to abnormal cardiovascular response at high altitude. Here, we reviewed the roles of PTMs in driving abnormal cardiovascular complications at high altitude.

## Introduction

Nowadays, a large number of lowlanders ascend to high altitudes (elevation ≥ 2,700 m) for military training, commercial working, mountaineering, and traveling every year. However, high-altitude environments, with the characteristics of lower barometric pressure, lower oxygen partial pressure, lower temperature, lower ambient humidity, and higher ultraviolet ray radiation, induce a series of system complications for the low-altitude human body (1, 2), especially for the cardiovascular system (3–5). Generally, people at low altitudes are prone to high altitude pulmonary edema (HAPE) due to acute hypoxia when suffering with rapid high-altitude exposure (2, 6). And lowlanders who live in a high altitude for a long time (> 3 months) are more likely to suffer from chronic high-altitude heart disease (HAHD), such as cardiac hypertrophy and pulmonary arterial hypertension (2, 6, 7). During acute and chronic high-altitude exposure in lowlanders, the ability of cardiovascular adaptation to high altitude is regarded as a key factor for the occurrence of abnormal cardiovascular complications. Therefore, effectively exploring relevant factors to evaluate the cardiovascular adaptation ability of lowlanders could be a promising strategy to decrease the occurrence of HAPE and HAHD.

Protein post-translational modifications (PTMs) refer to the covalent addition process of functional groups mediated by enzymes during or after protein translation (8). These modifications greatly increase the complexity of organisms and result in order-of-magnitude changes between the various proteins encoded in the genome and their biological functions (9, 10).

After translation, most proteins pass through different PTMs to maintain their structure, stability, and interactions with other actors in complex biological systems (11, 12). Moreover, transcription factors, signaling molecules proteins involved in hypoxia-induced myocardial cell proliferation, inflammation and reparation are subjected to various PTMs (13, 14). Therefore, the pathogenesis of dysregulation expression of enzymes in the PTMs steps is related to abnormal cardiovascular complications at high altitude.

Proteome-wide PTMs analysis in these high-altitude cardiovascular diseases will help clarify the underlying molecular mechanisms and provide new therapeutic targets for the prevention, diagnosis, and treatment of abnormal cardiovascular complications at high altitude. Here, we summarize recent findings on the roles of the most common types of PTMs in high-altitude cardiovascular complications, with emphasis on acetylation, phosphorylation, methylation, glycosylation, citrullination, crotonylation, lactylation, and sumoylation. In addition, we discussed the challenges and prospects of targeted PTMs application for abnormal cardiovascular responses at high altitude.

## High-altitude heart diseases

### The description of abnormal cardiovascular complications at high altitude

Apart from the original residents of the plateau for generations, high altitude could result in abnormal cardiovascular complications for low-altitude individuals. It may take days, weeks, and even months for people who previously lived at low-altitude to adapt to hypobaric hypoxia, thereby acute high-altitude sickness, with an incidence of 10–85% or chronic high-altitude sickness, with an incidence of nearly 100% may occur.

The abnormal adaptation response to high altitude can cause physio-pathological changes of varying organs and tissues. Specifically, it was reported that high altitude could induce irreversible/reversible damage to high oxygen and energy demanding tissues, such as the brain, heart, liver, gastrointestinal tract, and ocular tissue, over a certain period of time (15–20). The clinical characteristics commonly include blood pressure fluctuation, loss of memory function (declined cognitive function), absence of equilibrium function, headache, indigestion, decreased appetite, and so on (21–24).

As is well known, heart is one of the most metabolically active organs in the human body. In previous studies, much attention has paid to high altitude pulmonary edema (HAPE), a typical acute mountain sickness (AMS). They have been demonstrated to be implicated in acute altitude reactions for the reduction of partial oxygen pressure, and the possible mechanism may be related to uneven pulmonary vasoconstriction and disruption of the air-blood barrier, especially intracellular edema (25). Moreover, high altitude heart disease (HAHD) is used to describe the abnormal cardiovascular complications caused by high altitude. Generally, the narrow sense of HAHD refers in particular to high altitude pulmonary hypertension, a typical chronic high altitude disease. In a broad sense, HAHD refers to all kinds of heart damage caused by high altitude, including cardiac hypertrophy, arrhythmia, heart failure, pulmonary hypertension and so on. Evidences from professor Huang in Army Medical University considered that the pathogenesis of HAHD was related with abnormal cardiovascular adaptation response to high altitude (26).

### The process and evaluation of abnormal cardiovascular complications at high altitude

Acute cardiovascular caused by high altitude can often be divided into two typical processes. At initial entry to high altitude, cardiac output and heart rate are increased to compensate for low oxygen levels in the coronary artery. After a few days of good acclimatization, cardiac output returns to normal, mainly due to increased heart rate and reduced stroke volume. When inappropriate high-altitude adaptation occurs, ventricular filling patterns and ventricular systolic and diastolic functions would be impaired (27, 28). Then, sustaining hypoxia conditions may result in chronic cardiovascular complications, featured with hypoxic pulmonary vasoconstriction and the enhancement of pulmonary arterial pressure, which increases right ventricular afterload and impairs its function (28).

As for acute cardiovascular complications, signs (cyanosis, rapid heart and respiratory rates, edema of the face, liver enlargement, and rales) and symptoms (headache, dyspnea, cough, and sleeplessness) could be easily recognized. When it comes to chronic cardiovascular complications, there is no consensus on how to quickly identify them. Generally, the pulmonary artery pressure is used to evaluate the process of chronic cardiovascular complications. The international standard for chronic altitude disease is average resting pulmonary artery pressure > 30 mmHg or pulmonary artery systolic pressure > 50 mmHg, and average pulmonary artery pressure > 50 mmHg or pulmonary artery systolic pressure > 65 mmHg in infants. Additionally, the standard of pulmonary hypertension stipulated by the WHO is the mean pulmonary arterial pressure > 20 mmHg with capillary wedge pressure ≤ 15 mmHg.

Therefore, accurately predicting the risk level of abnormal cardiovascular responses at high altitude may be an effective way to reduce HAHD. More recently, cardiac function parameters at sea level were used as predictors of HAHD occurrence by screening echocardiographic parameters of the left ventricle, right ventricle, and pulmonary circulation (29). Moreover, changes in blood pressure (BP) and BP load were clinically found to be higher in HAHD patients, suggesting that BP load can be an effective indicator to evaluate cardiovascular adaptation ability (30). Furthermore, study found subjects with low 25% of the pulmonary volume values at low altitude were shown to be susceptible to high levels of pulmonary arterial pressure (31).

### Basic studies of abnormal cardiovascular complications at high altitude

At high altitude, the cardiovascular system must adapt to meet the metabolic need for oxygen (27). Meanwhile, it also increases the oxygen demand of the heart to react for the release of adrenaline and pulmonary artery pressure. It is well known that low-pressure hypoxia is a major challenge at high altitude and causes acute mountain sickness (29). Normal cardiovascular adaptation can effectively promote oxygen delivery to satisfy the body’s mitochondria metabolic demand and abnormal cardiovascular adaptation may lead to ventricular enlargement, pulmonary hypertension, and myocardial fibrosis. Animal studies found that mice exposed to a simulated high-altitude environment could develop myocardial hypertrophy and lesions involving the left and right ventricles (32). Electron microscopy showed dissolved or degenerated myofibrils, swollen mitochondria, dilated endoplasmic reticulum, and decreased glycogen granules (33).

Studies have shown that cardiac remodeling induced by altitude involved multiple mechanisms. Perinatal hypoxia at high altitude can cause neonatal pulmonary hypertension and right heart failure, which is closely related to the production of superoxide anion in mitochondria (34). Moreover, the high-altitude environment could destroy cardiac protein folding homeostasis and cause unbalanced endoplasmic reticulum stress. Animal studies showed that hypoxia stress enhanced the generation of free radicals, resulting in enhanced expression of hypoxia inducible factor 1α (HIF1α) (35). Combined with cold stress, reduced oxygen availability leads to extensive protein oxidative modification, accompanied by cardiac tissue damage and matrix remodeling. The presence of oxidized protein resulted in significantly up-regulated expression of Glucose Regulated Protein 78 (GRP78) and protein disulfide isomerase (PDI) in endoplasmic reticulum chaperone in hypoxia exposed animals (35).

## Post-translational modifications

### The basic descriptions of post-translational modifications

It is well-known that the human proteome is significantly more complex than the human genome. Specifically, researchers estimated that the human genome may contain between 20,000 and 25,000 genes, while the human proteome was estimated to contain more than 1 million proteins. The increase in complexity from the level of the genome to the proteome is further facilitated by protein PTMs. PTMs occur at different amino acid side chains or peptide bonds *via* covalently adding functional groups or proteins, proteolytic cleavage regulatory subunits, or entire protein degradation, and are usually mediated by enzyme activity.

PTMs are essential mechanisms to diversify protein functions, controlling protein stability, localization, and conformation. Moreover, PTMs can regulate protein interaction with other cellular molecules such as proteins, nucleic acids, lipids, and cofactors. Therefore, even if the expression level of the protein is not changed, the function of the protein can be significantly changed if the status of the PTMs is changed. Generally, the effect of PTMs on protein function is diverse, which is manifested in the following three aspects: (1) The same protein will be endowed with multiple functions even if only one type of modification occurs; (2) The same PTMs of the same protein can have different functions if it occurs on different amino acids; (3) The same protein may also have different modifications, and its functions and biological processes are more complex.

These modifications affect nearly all aspects of normal cell biology and pathogenesis. In fact, it is estimated that 5% of the proteome contains more than 400 PTMs. These enzymes include kinases, phosphatases, transferases, and ligases, which add or remove functional groups, proteins, lipids, or sugars from amino acid side chains. There are also proteases, which remove specific sequences or modulate subunits by breaking peptide bonds. Many proteins can also modify themselves using autocatalytic domains, such as self-kinases and self-proteolytic domains. Table 1 shows the most common and best studied types of PTMs, including acetylation, phosphorylation, methylation, glycosylation, citrullination, and sumoylation (36, 37). Recently, studies have shown that both bacteria and humans could adapt to new environments through PTMs (38–40), while the role of PTMs in abnormal cardiovascular adaptive response at high altitude has rarely been reported.

**TABLE 1:** Common types of post-translational modifications and target amino acid residues.

NO	Types of modification	Amino acid residues
1	Acetylation	Serine/threonine/alanine/lysine residues
2	Phosphorylation	Serine/threonine residues
3	Methylation	Lysine/cysteine residues
4	Glycosylation	Asparagine residue
5	Citrullination	Arginine residue
6	Crotonylation	Lysine residue
7	Lactylation	Lysine residue
8	Sumoylation	Lysine residue
9	Succinylation	Lysine residue

### The potential application area of post-translational modifications

The understanding of PTM function mainly focuses on phosphorylation, acetylation, ubiquitin, glycosylation, and other modifications. Even a basic understanding of these types of modifications would greatly expand our knowledge of biological processes and regulatory mechanisms. Therefore, the analysis of proteins and their PTMs is particularly important for the study of heart disease, cancer, neurodegenerative diseases, and diabetes.

Specifically, phosphorylation is involved in almost all biological processes. In addition to being the core mechanism of signal transduction, it is also involved in mitochondrial function, cytoskeleton regulation, cell membrane protein function and transcriptional regulation. And Non-histone acetylation is widely distributed in cytoplasm, mitochondria and other organelles, participating in signal transduction, energy metabolism, cytoskeleton, transcription factor activity, and so on. Additionally, glycosylation occurs mostly in proteins expressed on membranes and is important for macromolecular recognition. As for methylation, the methylation of DNA and histones is best known to be involved in epigenetic regulation. In recent years, more and more studies have found that methylation also occurs on non-histone proteins and plays an important role in many signal transduction processes. Interestingly, PTMs seem to play an important role in the process of heart remodeling induced by altitude. Table 2 shows the previous studies related with the PTMs patterns and high-altitude heart diseases.

**TABLE 2:** Post-translational modification patterns and loci of high altitude-related heart disease.

Heart disease related to the high altitude/Hypoxia	Type of TPMs	Modification site	References
High Altitude Pulmonary Edema (HAPE)	Acetylation/Methylation Phosphorylation	Histone modification/DNA methylation Vascular endothelial cadherin/Na, K-ATPase	41 42, 43
High Altitude Pulmonary Hypertension (HAPH)	Methylation Glycosylation Acetylation Phosphorylation Ubiquitination	N6-methyladenosine *O-*GlcNAcylation Histone 4 MLC20/MYPT1/eNOS HIF2α	44 45 46, 47 48–51 52
Right Ventricular Hypertrophy	Methylation Carbonylation Oxidative modifications NA Carbonylation	Lysine 36 on histone 3/N6-methyladenine NA SU5416/ovalbumin Sodium-calcium exchange current Annexin A1	53 54 54 55 56
Left Ventricular Hypertrophy	Methylation Carbonylation	Lysine 36 on histone 3/N6-methyladenine Annexin A1	53 56
Arrhythmia	NA	Sodium-calcium exchange current	55
Ischemia Reperfusion Injury (I/R)	Succinylation Sumoylation	P53 HIF1α	57 58

PTM is an important regulatory mechanism of biological functions, as important as transcription and protein expression regulation, but much more complex. However, the current study is only the tip of the iceberg when it comes to the profound understanding of PTMs.

## Role of different post-translational modifications in high-altitude cardiac remodeling

### Acetylation/deacetylation

Protein acetylation/deacetylation is a reversible PTM in which an acetyl group is added or removed from the amino acid of a protein (59, 60). The process is catalyzed by two groups of enzymes, K lysine acetyltransferase (KAT) and K lysine deacetyltransferase (KADC) (59–61). Acetylation or deacetylation of non-histone proteins modifies a series of biological processes including enzymatic activity, inflammation, autophagy, protein–protein interactions, and protein localization. Either aberrant expression of acetyltransferases/deacetylases or alteration in the status of acetylation of protein targets are found in acute and chronic effects of high altitude on the cardiovascular system of lowlanders.

Sirtuin (SIRT) is a mammalian NAD^+^-dependent deacetylase family composed of seven SIRT1-7 members, which plays an important role in the abnormal cardiovascular complications at high altitude (62). Recently, the potential mechanisms of SIRTS in HAHD have been extensively investigated from clinical observations to molecular studies (63, 64). Animal studies have shown that SIRT1 expression was reduced in an acute hypoxia-induced pulmonary hypertension rat model. Interestingly, resveratrol (a small molecule agonists of SIRT1) and SRT1720 (a selective activator of SIRT1) could reverse the proliferation of pulmonary smooth muscle cells (65). Moreover, cell studies confirmed that SIRT1 could promote pulmonary artery endothelial cells proliferation and inhibit apoptosis in a simulated hypoxic environment. This mechanism may be related to the activation of protein kinase B (Akt) signaling pathway and B-cell lymphoma-2 (Bcl-2) pathway (66). Moreover, it is well-known that cardiovascular complications are often accompanied with mitochondrial dysfunction (67), and acetylation/deacetylation of mitochondrial proteins are considered as a key regulatory factor of mitochondrial metabolism and function (68). SIRT3 has been reported to be a major mechanism for regulating protein acetylation in mitochondria through pyruvate dehydrogenase and aconitase deacetylation (68). Overexpression of SIRT3 has been shown to prevent the accumulation of reactive oxygen species (ROS) in cardiomyocytes in response to different environmental stressors (69) and α1-adrenergic receptor agonist phenylephrine (70).

Cardiomyopathy hypertrophy is a typical abnormal cardiovascular complication of high altitude, ultimately leading to heart failure (71, 72). Histone acetylation plays an important role in epigenetic remodeling in the pathogenesis of cardiac hypertrophy (73). Specifically, histone deacetylase 2 (HDAC2) can result in severe cardiac hypertrophy, while HDAC2 knockout mice are resistant to exogenous hypertrophy stimulation (73, 74). Additionally, HDAC1 and HDAC5 were elevated in the right ventricles of rats when exposed to acute hypoxia. Valproic acid and class I HDAC inhibitors SAHA could alleviate and reduce the development of hypoxia induced pulmonary hypertension (75).

The role of other members of the histone deacetylase family in PTMs in abnormal cardiovascular complications at high altitude is unclear, but the potential therapeutic effects of SIRT1 agonists or class I histone deacetylase inhibitors have been observed in existing studies. Therefore, histone acetylation and deacetylation modification may be one of the options for the prevention of acute and chronic cardiovascular complications at high altitude.

### Phosphorylation

Protein phosphorylation is the addition of a phosphate group to an intermediate metabolite or protein. Enzymes capable of removing phosphate groups are called phosphatases (76). Protein phosphorylation occurs on many types of amino acids (the main unit of proteins), with serine predominating, followed by threonine. Dephosphorylation refers to the removal of phosphate groups and acts as an “on/off” effect for many organisms (77).

**FIGURE 1:**
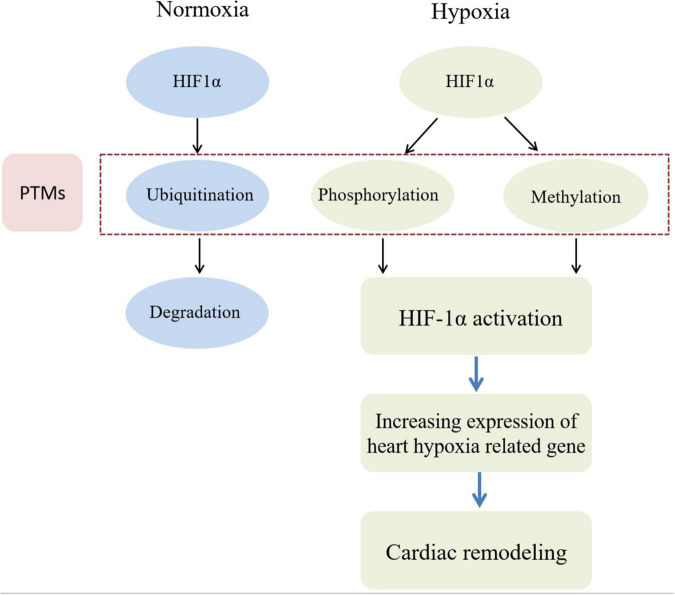
HIF phosphorylation and methylation are involved in cardiac remodeling induced by hypoxia. In the normoxia condition, HIF1α protein in myocardial cell can be degraded by ubiquitin modification. However, in the hypoxia condition, HIF1α protein in myocardial cell be activated by phosphorylation and methylation modification. Then heart hypoxia related down-stream genes can be cascade activated to induce myocardial cell abnormal proliferation. Finally, cardiac remodeling would be occurred during sustaining chronic hypoxia conditions.

It is widely believed that metabolic changes in individuals exposed to high altitude are due to ambient hypoxia and lower atmospheric pressure. The discovery of hypoxia inducible factor 1 (HIF1), a transcription factor, is a breakthrough in the study of high-altitude adaptation response (78). A recent study found that HIF1α transcription primarily regulated metabolic reprogramming, while HIF2α exerted a greater role in regulating angiogenic extracellular signaling, guidance cues, and extracellular matrix remodeling factors (79). HIF1 is a heterodimer composed of oxygen-sensitive HIF1α and oxygen-independent subunit HIF1β. Recently, a key role of PTM of HIF1α in cellular oxygen sensitivity has been identified (80). Under normoxic conditions, HIF1α hydroxylates on specific proline residues, leading to immediate ubiquitination and subsequent proteasome degradation of α subunits (81). In addition, hydroxylation of asparagine residues blocks transcriptional activity of HIF1 by inhibiting its interaction with coactivators. Conversely, the elimination of proline hydroxylation leads to HIF1α stabilization under hypoxia, while the absence of asparagine hydroxylation allows transcriptional activity. In addition, transcriptional activity can be regulated by phosphorylation or methylation modification of HIF1 (81). Figure 1 shows the different PTMs of HIF1α under normoxia and hypoxia conditions, respectively.

Janus kinase 2 (JAK2) was thought to be involved in pulmonary vascular remodeling in pulmonary hypertension. In response to hypoxia-induced activation, JAK2 phosphorylates signal transduction and transcriptional activator 3 (STAT3), which then binds to the Cyclin A2 (CCNA2) promoter to transcription cyclin A2 expression. And JAK2 inhibitor could alleviate hypoxia-induced proliferation of primary human pulmonary artery smooth muscle cells (PASMC) (82). Endothelial dysfunction plays an integral role in HAHD, and AMPK (AMP-activated protein kinase) and ACE2 (angiotensin-converting enzyme 2) are crucial in endothelial homeostasis. Phosphorylation of ACE2 by AMPK can enhance the stability of ACE2, which increased Ang (angiotensin) 1–7 and endothelial nitric oxide synthase-derived NO bioavailability (83).

### Methylation

The best well-known methylations are DNA and histones modifications, mainly involved in epigenetic regulation. In recent years, more and more studies have found that methylation also occurs on non-histone proteins and played an important role in various signal transduction processes. Protein methylation is a multifunctional PTM involving a variety of major cellular processes. Methylation can modulate protein activity, stability, localization, and/or interactions, resulting in specific downstream signaling and biological outcomes. Lysine methylation is a dynamic and fine-tuned process, and its irregularities often lead to human pathology (84). HIF1α and HIF2α are the main regulators of cellular responses to hypoxia. PTMs of HIF1α and 2α are required to regulate their function. The methylation of non-histones by the SET domain containing lysine methyltransferase Set7 is a novel mechanism for regulating the function of cellular proteins under various cellular stresses (85). Xing Liu et al. found that Set7 induced HIF1α methylation at lysine 32 and HIF2α methylation at lysine K29. This methylation inhibited HIF1α/2α expression by interfering with the hypoxia response element of HIFα in the promoter of the HIF target gene (85). Moreover, Xiong et al. found that yaks coped with high altitude hypoxia stress by changing the expression of methylation of hypoxia-inducing factors (86). This adaptation has been observed in highland animals and humans (87–90).

### Glycosylation

Protein glycosylation is one of the most complex forms of PTMs. Glycosylation is a process in which sugar chain molecules are transferred to proteins and special amino acid residues on proteins form glycosidic bonds under a series of enzyme regulation actions (91). Glycosyltransferases and glycosidases are responsible for catalyzing the transfer of glycosyl-based residues from donor to recipient proteins and the removal of glucose residues from oligosaccharide complexes. Studies have shown that 70% of human proteins contain one or more sugar chains, and 1% of the human genome is involved in the synthesis and modification of sugar chains. Among the various types of glycosylation identified, *O-*linked *N-*acetylglucosamine (*O-*GlcNAcylation) and *N-*glycosylation are the two most common types (92, 93).

It is well known that abnormal glycosylation and impaired glycosyltransferase localization and expression are closely related to the occurrence and development of various hypoxia diseases, including high altitude polycythemia (94), hippocampal neuron damage during chronic hypoxia (95), and brain injury from intracerebral hemorrhage (96). Recently, it has been found that the transmembrane glycoprotein receptor CD147 played an important role in the induction of hypoxia-induced cardiac remodeling. Glycosylated CD147 could significantly attenuate stress-induced pathological cardiac remodeling, accompanied by decreased oxidative stress and ferroptosis (97). Arrhythmia is proved be a typical abnormal cardiovascular complication in high altitude. Ufret-vincenty, C. A. investigated the role of ion channels in arrhythmias and eventually found that deficient glycosylation of Na^+^ channel contributed to Na^+^ current-dependent arrhythmogenesis in heart failure (98). Moreover, hypoxia-induced pulmonary hypertension is a progressive disease mainly caused by long-term exposure at high altitude (99). Increased pulmonary vascular resistance and pulmonary artery pressure results in enhancement of right ventricular afterload, leading to right heart failure. Further studies have shown that mitochondrial dysfunction played a key role in high-altitude pulmonary hypertension. Protein *O-*GlcNAcylation protected heart tissues by attenuating the formation of mitochondrial permeability transition pores (mPTP) and subsequent loss of mitochondrial membrane potential (100). Although much progress has been made in the role of glycosylation in abnormal cardiovascular complications at altitude, the role of different glycosyltransferases in HAHD remains to be further investigated.

### Sumoylation

Sumoylation is the process of covalently attaching ubiquitin associated modification small molecules to multiple proteins to regulate their function (101). The Sumoylation of proteins has become an important PTM that modulated cellular responses to different types of stress, including hypoxia, cold, and oxidative stress. Previous studies have shown that ubiquitination and deubiquitination were closely related to tumor invasion and escape (102). In colon cancer and breast cancer, tumor suppressor gene P27 is degraded by ubiquitination, which increases tumor aggressiveness. Conversely, deubiquitination stabilizes tumor suppressor genes and thus inhibits tumor progression (103).

In the hypoxia induced pulmonary arterial hypertension mouse model, SUMO1 expression was significantly increased, which was associated with autophagy activation, pulmonary artery vascular smooth muscle cells dedifferentiation, and pulmonary vascular remodeling. Moreover, SUMO1 knockdown reversed hypoxia-induced pulmonary artery vascular smooth muscle cells proliferation and migration (104). Intact mitochondrial homeostasis has been shown to be critical for cardiac systolic function and cardiomyocyte metabolism, and dynamic-associated protein 1 (Drp1) is a key factor in maintaining mitochondrial homeostasis (105). Studies have shown that Drp1 was affected by many PTMs, including ubiquitination. It was found that Drp1 could be ubiquitinated by Parkin, and Parkin targeted Drp1 for proteasome degradation, thereby affecting the process of mitochondrial fission and fusion (106).

### Citrullination

Citrullination refers to the conversion of arginine residues to citrulline residues on the protein peptide chain under the action of protein arginine decarboxylase (107). These modifications are most commonly found in rheumatoid arthritis (RA) and cancer (108). Arginine deamination (also known as citrullination) plays a major role in the progression of rheumatoid arthritis by producing autoantibodies and exacerbating inflammatory responses (109, 110). Studies have shown that citrulline regulates cell apoptosis and differentiation, promotes epithelial–mesenchymal transition and metastasis, and the potential application of citrulline antigen in immunotherapy. Citrullination is also used as a cancer biomarker (109, 111). Currently, the role of citrulline modification in abnormal cardiovascular complications at high altitude has not been reported, but we believe that with the further study, the role of citrulline modification in abnormal cardiovascular complications at high altitude will be gradually discovered.

### Succinylation

Back in 2010, the University of Chicago team first discovered the PTMs of succinylation of lysine (112). Compared with methylation and acetylation, lysine succinylation induced more changes in protein properties (113). This is because the succinylation is given two negative charges, the valence state changes from + 1 to − 1, which is higher than the charge changes caused by acetylation (+ 1 to 0) and mono-methylation (no change). In addition, succinylation leads to larger group structures that are more capable of altering protein structure and function (112). Meanwhile, succinyl-CoA is a cofactor of the enzyme that regulates succinylation. As an important intermediate product of metabolic reactions, succinyl-CoA appears in tricarboxylic acid (TCA) cycle, the synthesis of porphyrins and the decomposition of some branched amino acids (114, 115). Its stable state is essential for maintaining normal physiological activities of cells. Genetic mutations in succinyl-CoA metabolism are likely to cause diseases. During the neonatal period, significant maturation changes occur in cardiac energy metabolism, from glycolysis to fatty acid oxidation. Acetylation and succinylation of lysine residues are novel PTM that controls cardiac energy metabolism (116). Researchers investigated the effects of protein succinylation on cardiac energy metabolic maturation at 1, 7, and 21 days of age in rabbits. The results showed that the rate of fatty acid β-oxidation increased at 21 days of age, and the rate of glycolysis and glucose oxidation decreased. The degree of acetylation of fatty acid oxidase, long-chain acyl-CoA dehydrogenase and β-hydroxyacyl-CoA dehydrogenase was positively correlated with their activity and fatty acid β-oxidation rate (117). Sadhukhan et al. analyzed acyl-CoA molecules in different mouse tissues and found that different tissues had different acyl-CoA profiles. Succinyl-CoA is the most abundant acyl-CoA molecule in the heart, and the succinylation of myocardial lysine is regulated by SIRT5 (118). Considering the disorder of cardiometabolic at high altitude environments, protein succinylation needs to be further explored.

### Lactylation

Lactylation modification is a newly discovered PTM, first described by Zhang et al. and published in Nature (119). Using a bacterial exposed M1 macrophage model system, researchers demonstrated that histone lactation and acetylation have different temporal dynamics. The results suggested that elevated histone lactate levels induced homeostasis genes, including arginase 1, to participate in wound healing during late polarization of M1 macrophages (120). Hideo Hagihara described the presence of lactate modifications in neurons that affected neural excitability (121). High altitude and lack of oxygen also lead to increased glycolysis and lactic acid production. At present, it is not clear whether excessive lactic acid can cause protein lactylation modification. Hence, systematically exploring the proteins lactylation modification at high-altitude hypobaric hypoxic environment could be a promising way to expand the mechanism of abnormal cardiovascular complications at high altitude.

### Crotonylation

The Crotonylation of histone lysine residues was first identified as being enriched in promoter and enhancer regions of human male reproductive cells (122). Subsequently, non-histone crotonylation was found to be particularly enriched in nucleoproteins involved in RNA processing, nucleic acid metabolism, and chromosomal tissue (123). More studies identified lysine crotonylation (Kcr) in non-histone proteins (124–126). Kcr is conserved and regulated by a range of enzymes and coenzymes, including lysine crotonyltransferase (writer), lysine decarboxylase (eraser), certain YEATS proteins (reader), and crotonyl-coenzyme A (donor) (127). It is well known that P53 is a tumor suppressor protein that binds to specific DNA sequences and transcriptionally activates target genes to regulate critical cellular processes, including cell cycle control, apoptosis, and DNA repair under genotoxic stress. Meanwhile, P53 is also involved in cardiac remodeling induced by altitude hypoxia (128). These results suggest that PTM of P53 signaling pathway may play an important role in high altitude adaptation response (129).

## Therapeutics targeting post-translational modifications in the treatment of high-altitude heart disease

**FIGURE 2:**
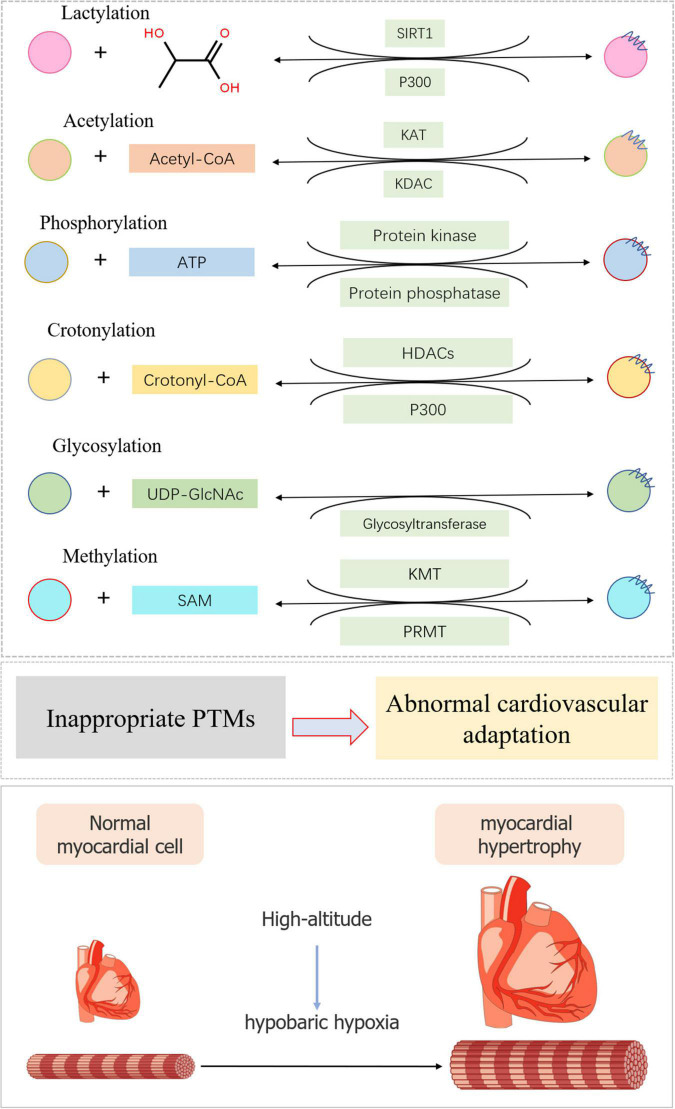
Typical post-translational modifications in high-altitude cardiovascular complications. The substrate and modification enzyme of lactylation, acetylation, phosphorylation, crotonylation, glycosylation, and methylation were exhibited using a diagrammatic plan. When inappropriate PTMs occurred, myocardial cell would become hypertrophic during high-altitude accommodations mainly due to hypobaric hypoxia.

A series of studies revealed the role of PTMs in abnormal cardiovascular complications at high altitude, providing a basis for the further prevention, diagnosis, and treatment of PTMs in altitude heart disease (Figure 2).

Specifically, analysis of cells and tissues isolated from *in vivo* preclinical models of pulmonary arterial hypertension and human pulmonary hypertension patients revealed significant changes in the expression levels of various HDACs, SIRT1 and SIRT3 proteins. The roles were demonstrated to be associated with proliferative, inflammatory, and fibrotic phenotypes, and cardiac remodeling processes (59, 61, 69, 130). Due to the reversible ability of PTM acetylation, the efficacy of a number of small molecule inhibitors, such as vorinorestat, valproic acid, sodium butyrate, and resveratrol, have been evaluated in various preclinical models of cardiovascular diseases. This suggests the therapeutic value of targeted histone acetylation pathways in hypoxia-induced pulmonary hypertension and right heart failure induced by pulmonary hypertension (131).

In recent years, high-altitude response caused by short-term intermittent hypoxia exposure has attracted attention again (132). One of the mechanisms of altitude adaptation caused by intermittent hypoxia exposure is proteins PTMs. HIF1 is a major regulator of cellular and systemic oxygen homeostasis. HIF1α is methylated or phosphorylated under intermittent hypoxia induction, activating cardiac genes that encode proteins involved in hypoxia homeostasis response and participate in altitude adaptation. Other types of PTMs, such as methylation, crotonylation, and lactation, have also shown potential therapeutic effects. However, these treatments were still in the research stages and have not been applied to clinical practice.

## Conclusion

In this review, we discussed the main types of PTMs in high-altitude cardiovascular adaptation response, including acetylation, phosphorylation, methylation, glycosylation, sumoylation, citrullination, succinylation, lactylation, and crotonylation. Many PTMs have been discovered in recent years, and the key role of PTMs has attracted extensive attention from researchers due to the discovery of enzymology, function and mechanism. In addition, with the advent of new proteomic techniques, new PTMs have been shown to play essential roles in hypoxia-induced. However, the role of PTMs in myocardial remodeling, such as lactylation, citrullination, and crotonylation, has not been fully appreciated and required further investigations. We believe that as research continues, the role of PTMs in abnormal cardiovascular complications at high altitude will become clear.

## Author contributions

HD and ZZ conceived and designed the review. JH, SX, and XW analyzed literatures. HL and LC collected the literatures. PL and HL validated the method and literatures. JH and PL wrote this manuscript. All authors read and approved the final manuscript.

## Abbreviations

ACE2: Angiotensin-converting enzyme 2
α -SMA: Alpha-smooth muscle actin
Akt: Protein Kinase B
AMPK: AMP-activated protein kinase
Ang: Angiotensin
ATG14: Recombinant Human Autophagy Related 14
COA: Coenzyme A
Drp1: Dynamic-associated protein 1
GSK3: Glycogen synthase kinase 3
HAHD: High-altitude heart disease
HDAC2: Histone Deacetylases 2
HIF1: Hypoxia inducible factor 1
HPH: Hypoxia-induced pulmonary hypertension
Inpp5f: Inositol polyphosphate-5-phosphatase F
IHC: Immunohistochemistry
JAK2: Janus kinase 2
KADC: Lysine Deacetylase
KAT: Lysine acetyltransferase
Kcr: Lysine crotonylation
PTMs: Post-translational modifications
LC3: Light chain 3
mPTP: mitochondrial Permeability Transition Pore
NDDs: Neurodegenerative disorders
NO: Nitric oxide
PASMC: Pulmonary arterial smooth muscle cell
PH: Pulmonary hypertension
PI3K: Phosphatidylinositol 3-kinase
RA: Rheumatoid arthritis
ROS: Reactive oxygen species
SIRT1: Silent mating type information regulation 2 homolog 1
SIRT3: Silent mating type information regulation 2 homolog 3
SIRT5: Silent mating type information regulation 2 homolog 5
SM22: Smooth muscle 22
SM-MHC: Smooth muscle-Major histocompatibility complex
STAT3: Signal transduction and transcriptional activator 3
SUMO: Small ubiquitin-associated modified molecules
TAC: Tricarboxylic acid cycle
TP: Triptolide
VSMCs: Vascular smooth muscle cells.
